# Tall Oaks from Little Acorns

**Published:** 1887-02

**Authors:** 


### TALL OAKS FROM LITTLE ACORNS.

(EIGHTH ILLINOIS REPORT, S. B. HEALTH.)

In 1877 the first medical bill passed the Illinois Legislature, creating a State Board of Health. For awhile the functions of the board were limited to the protection of the public health from adulterated food and drink, and from unsanitary surroundings and habits, etc., and from the introduction of infectious diseases, etc. It dealt with quarantine, local inspections, and vital statistics. Bye and bye through the instrumentality of that heroic worker in the cause of higher education, Dr. J. H. Rauch, the distinguished Secretary, it was gotten through the heads of the legislators that in the exercise of police measures whereby public health is protected, it is as much the duty of the State to protect its citizens from injuries they may sustain from the practice of incompetent physicians and surgeons as from any other source of danger—and thus the scope of the board's influence and powers—long limited to the agency of the State in matters of hygiene beyond the control of the individual,was extended to embrace measures of preventive medicine in general—and finally broadened so as to include within its scope the field of curative medicine, which led necessarily to the control, by the State, of the practice. The legislators were made to see as Dr.

Rauch expressed it, that "the authority upon which is based the right of the state to regulate the practice of medicine is the inherent and plenary power which resides in the State, to prohibit all things hurtful, and to promote all things helpful ; to the comfort, welfare and safety of society. Thus the medical practice act in Illinois was primarily a police regulation, intended to rid the State of quacks, and incidently it has become educational, since, to carry out the principles just stated, the State must see that those who are permitted to practice. are educated and qualified to practice, and, it became a part of the duty of the Board of Health in Illinois to carry out the provision of the act. It is well-known that in consequence of reforms instituted by this board in the matter of requirements to matriculate in any medical college, the standard of medical education throughout America has been considerably raised. This was done by the board's assuming to fix a standard to which college swere required to conform, in graduating students; those failing to do so were taboed, rated as not "in good standing" and their diplomas were not recognized. This had the effect of causing a majority of medical colleges to advance their standard of education, 'till now, most schools require a three years graded course, and the days of easy graduation, and cheap diplomas are over.

Thus tall oaks from little acorns grow, and thus it ought to be in all states; and thus it will be in Texas, if our legislators can be made to take a broad and comprehensive view of State Medicine." They must be made to see and acknowledge the want of a State Board of Health, and that the functions of that board—its mere police functions—the conservation of public health,—necessarily extend to protection from the quack, as well as from epidemics ; from the ignorant prescriber of deadly medicines, as from the dangers of adulterated food; from the butchery of so-called "phenomenal surgeons," phenomenal in monstrosity and in lying promises—a sense little contemplated by some who have recently disgraced Austin by their tricks and self-laudation—as from the typhoid infection of sepe-water, privy-vault–contaminated-wells ; as much from the licensed "family physician" who kills with impunity, because he does not know the dose of morphine, as from any other source of danger, and more, because this danger travels under the guise of safety, and patient are lured to destruction, whereas other dangers may be seen, and perhaps avoided.

But the Illinois bill is still defective, as evidenced by the decision of a court recently, were a violator of the law escaped on a technicality, and Dr. Rauch writes us he is endeavoring to get it improved.

There is now a bill to create a State Board of Health for Texas (not a bill to regulate the practice) in the hands of a member to be presented to the legislature now in session. It was drafted, we learn, by the committee on legislation appointed by the Texas State Medical Association. We look to see it share the fate of all its predecessors. But, it is a mere matter of time; the quack must go. We are only planting, or trying to plant the acorn; the oak will come later.

Some of the above facts are gleaned from the Eighth Annual Report of the Illinois State Board of Health, for a copy of which we are indebted to the energetic Secretary, Dr. J. H. Rauch. It is exhaustive, and treats, in detail, of everything which comes within the jurisdiction of a board of health.

